# Abstracts and Gleanings

**Published:** 1887-12

**Authors:** 


### ABSTRACTS AND GLEANINGS.

**Antipyrine.**—The application of antipyrine seems to extend day by day. Professor Germain See is one of its decided partisans in its use against pain, and goes so far as to count on it in the place of morphine Its easy solubility allows of its use in subcutaneous injections, and Dr. Sée adopts this form for rheumatic pains in half-gram doses. It must be stated, however, that at the same time three grams are given by the mouth, with the result of nearly always calming the pain both in chronic and acute gout.

M. See also states that he cured three cases of tic-douleureux, and also cases of painful zona, lumbago, megrim, hapatic, colic, nephritic colic, angina pectoris, asthma, and a long list of other troubles, including heart-pains. Professor See does not hesitate to conclude that it can entirely replace morphine, and certainly it has not the inconveniences of that drug; but will it always have the fiedelity of action that it has against pain? Time alone can tell. At the present moment all the great hospital services are trying antipyrine in all sorts of troubles, so that in a few months its remarkable sedative influence will be investigated enough to enable us to report more fully upon it.

M. Chouppe reports to the Societe de Biologie that he had occasion to employ antipyrine in rectal injections to calm uterine colic. In one case a woman was suffering with intense after-birth pains, and an injection containing one gram of the drug removed the pain. It returned after several hours, but a second injection was given with the result of a definite cure. A second observation was upon a woman, who, for several years, had violent colic at every menstrual period, which lasted several hours at a time. Relief could not be obtained without great difficulty by the use of doses of laudanum or chloral large enough to produce profound sleep. At her last menstrual period, during a most violent attack of pain, one gram of antipyrine was given by rectal injection, with the result of complete and definite calm being established within a quarter of an hour.

The same author also spoke of the "Reciprocal Action of Antipyrine and Strychnine." He made a number of experiments to see if antipyrine, in large doses, would modify the form and intensity of strychnine convulsions, according to a suggestion of Professor Brown Sequard. He found that the convulsions produced in animals by antipyrine did not at all resemble those of strychnine in three important points: (1) They were not brought on by peripheric excitation; (2) their form was not so tetanic; they consisted of a series of rapid clonic convulsions without any real tetanization of the muscles; (3) they did not act so much on the muscles of respiration, and this function was not at all suspended with danger of asphyxia, as in strychnine convulsions. Adding the action of strychnine to antipyrine, M. Chouppe injected into the viens of an animal which was already in a state of convulsion from antipyrine a dose of strychnine that should have killed it, but the antipyrine convulsions were simply replaced by strychnine. Then a stronger dose of antipyrine was injected into its veins, which caused the strychnine form to give way to the antipyrine convulsions. The result of various experiments seems to establish that the action of antipyrine to some extent prevents the convulsions of strychnine by reducing the power of the spinal marrow.—*Philadelphia Medical Times.*

**The Indications and Rationale in Washing Out the Puerperal Uterus.**—Dr. J. Halliday Croom recently read a paper before the Edinburgh Obstetrical Society in which he formulates the circumstances under which the antiseptic washing out of the uterine cavity is indicated (*Obstetric Gazette*, August, 1887).

*Indications.*—Where, with localized tenderness over the uterus, there is a high pulse and temperature, and a fetid discharge. It is to be observed that the discharge must be fetid *from the uterus.* In order to decide this question, it is essential to wash out the vagina with an antiseptic wash—inodorous, such as corrosive sublimate—and then putting the finger up and into the cervix, to decide whether it is fetid or not. As the author points out in the sequel, all first washings out should be performed under chloroform, therefore he always explores the cavity of the uterus with the finger. No reference need here be made as to the ease with which this can be performed, at least during the first week of the puerperium. Even at a long period after labor, the carbolized fingers can be comparatively easily introduced. In most cases some morbid product will be found, and in all cases the necessary dilatation of the cervix will much facilitate the process of washing out.

2. Where, with a high pulse and temperature, there is any question as to the absolute complete delivery of the placenta; and, in this connection, it is impossible to emphasize too strongly the importance of examining closely the placenta after delivery, whether it be expressed, extracted, or delivered spontaneously. Such care will often eliminate at once any possible cause of infection.

3. Where portions of membrane have been retained *in utero*, and give rise to increase in pulse and temperature. Here, however, it should be noticed that it is possible to do harm in endeavoring to remove the membranes completely at the time of delivery. It is much better to leave a portion of membrane than to open up the genital canal in search for a small piece.

4. After the birth of a putrid fœtus.

5. Where the uterus remains abnormally large after labor, and where, as a result, owing to the presence of decomposing clot, symptoms of septic infection develop themselves. In such cases, washing out ought to be accompanied by the introduction of the finger within the uterus, and in all such cases quinine ought to be administered in large doses.

6. In cases where, late on in the puerperium, symptoms of septicæmia develop themselves.

7. In those somewhat rare, but well-recognized cases, where, from acute flexion of the uterus, the lochia are retained and decompose.

8. In some cases of imperfect abortion and premature labor, and in all cases where the uterus, under such circumstances, has curetted.

9. In all cases where the hand has been introduced—say in cases of post-partum hemorrhage, adherent placenta, or uterine hydatids—washing out the uterus with hot antiseptic water is the recognized treatment.

*Rationale.*—"What is the *rationale*," the author next asks, "of washing out the puerperal uterus in septicæmia?" It seems, at first sight, open to doubt how far washing out the uterine cavity can prove effective in checking septicæmia if rapidly-multiplying microbes have already passed into the system.—*Ther. Gazette.*

**Positions that Affect Sleep.**—According to Dr. Granville the position affects sleep.

A constrained position generally prevents repose, while a comfortable one wooes sleep.

He says:

"Lying flat on the back, with the limbs relaxed, would seem to secure the greatest amount of rest for the muscular system.

"This is the position assumed in the most exhausting diseases, and it is generally hailed as a token of revival when a patient voluntarily turns on the side; but there are several disadvantages in the supine posture which impair or embarrass sleep.

"Thus in weakly states of the heart and blood-vessels and certain morbid conditions of the brain, the blood seems to gravitate to the back of the head and to produce troublesome dreams.

"In persons who habitually, in their gate or walk, stoop, there is probably some distress consequent on straightening the spine.

"Those who have contracted chests, especially persons who have had pleurisy and retain adhesions of the lungs, do not sleep well on the back.

"Nearly all who are inclined to snore do so in that position because the soft palate and uvula hang on the tongue, and that organ falls back so as to partly close the top of the wind-pipe.

"It is better, therefore, to lie on the side. and in the absence of special diseases rendering it desirable to lie on the weak side, so as to leave the healthy lung free to expand, it is well to use the right side, because when the body is thus placed the food gravitates more easily out of the stomach into the intestines, and the weight of the stomach does not compress the upper portion of the intestines.

"A glance at any plate of the visceral anatomy will show this must be.

"Many persons are deaf in one ear and prefer to lie on a particular side; but, if possible, the right side should be chosen.

"Again, sleeping with the arms thrown over the head is to be deprecated; but this position is often assumed during sleep, because circulation is then free in the extremities, and the head and neck and muscles of the chest, are drawn up and fixed by the shoulders, and thus the expansion of the thorax is easy.

"The chief objections to these positions are that they create a tendency to cramp and cold in the arms, and sometimes seem to cause headaches during sleep, and dreams.

"These small matters often make or mar comfort in sleeping." —*Pacific Record.*

**Experiments with Antifebrin.**—According to the Vienna correspondent of the *Medical Press*, July 27, 1887, Dr. Weinstein summarizes the results which he had obtained in his experiments with "antifebrin" in the clinics of the General Hospital in the following way: As an antipyretic from $\frac{1}{8}$ to $\frac{1}{4}$ gramme dose is sufficient to lower the high temperature by three degrees after the lapse of from two to four hours. Antifebrin may be administered in daily doses of from 1 to 2 grammes. 2. The transition of the high temperature to the lower, and *vice versa*, is a gradual one. The apyrexia usually lasts for some hours, sometimes from twelve to sixteen. 3. No bad consequences supervene when the drug is administered with precaution. After large doses, such as 0.25 or 0.50 gramme, have been administered at one time, great perspiration, and occasiona ly also shivering or cyanosis, used to occur. Hyperpyresis and collapses hardly ever supervened. 4. The blood-pressure first shows a tendency to increase, but is no longer influenced when the antifebrin has been administered for a longer interval of time. The rigidity of the blood-vessels increases slightly. 5. The best results were obtained with antifebrin in cases of erysipelas, puerperal process, and intermittent fever. The swelling of the spleen decreased. Dr. Weinstein remarks that antifebrin is best fitted for regulating the temperature, for producing a gradual decrease of the abnormal warmth of the blood, so that no collapse may occur. He advises its administration in doses of from $\frac{1}{8}$ to $\frac{1}{4}$ of a gramme (2 to 4 grs.), from three to four times daily, and the drug is to be administered only when the temperature has reached 40° C. and over. A decrease of 2° C. is sufficient. The administration of the antifebrin as an antirheumatic is to be recommended in acute cases, in doses of $\frac{3}{4}$ of a gramme at each two hours, and of from 1 to $1\frac{1}{2}$ grammes daily. The antifebrin may be administered in the same doses as a "nervinum," but only with a palliative effect.—*Ther. Gazette.*

**The Treatment of Chronic Abscess by Injections of an Ethereal Solution of Iodoform.**—Terchere (*Rev. de Chir.*, June, 1886) reports twenty-three cases which were treated in this manner, and gives the following directions in regard to the operation: The solutions of idoform should be of varying strength, one of 5-per cent. being used for large abscesses, and one of 10-per cent. for small ones, while small, superficial abscesses may be filled with a saturated solution. If the skin over the abscess is not affected, the needle of hypodermic syringe is introduced in an oblique direction, so as to form a valvular fold; the pus is then drawn off, and the iodoform solution is injected. If, however, the skin over the abscess is quite thin, the pus is removed with an aspirator, and the opening made by the needle is sealed with collodion, after which a hypodermic syringe needle is inserted into the abscess cavity, and the injection is made as before. The object of these manœuvres is to prevent the ether from escaping through the puncture, as it at once tends to do on becoming volatilized. As the solution volatilizes, the iodoform deposits over the entire inner surface of the abscess, and is slowly absorbed—so slowly, in fact, that the danger of poisoning by the drug is said to be very slight. The phenomena observed after an injection are, briefly, as follows: Rapid and sometimes excessive swelling results from the volatilization of the ether, but this soon subsides. If the skin over the abscess is healthy, the abscess cavity will speedily be replaced by indurated tissue, without the occurrence of any external change. If the skin is already inflamed, it will separate in a few days in the form of a yellowish slough, after which healing will occur by granulation, the resulting cicatrix being slight. The advantages alleged for this method of treatment are the perfect safety of the operation, the rapidity of the cure, the fact that the patient is not confined to his bed during the treatment, and the non-recurrence of the abscess.—*Canada Medical Record.*

**A Case of Traumatic Tetanus Cured.**—Dr. Mayer makes the following communication to the *Altegemeine med. Central-Zeitung*, August 6th, 1887 :

P. Reckert, nineteen years of old, of strong physique, wantonly wounded himself on the point of his left forefinger in trying to break a china plate with his hands. On the evening of the third day after the wounding, the patient noticed, after being wet through by a cold rain, that he could no longer open his mouth. As this was accompanied during the night by painful contractions of the muscles of the neck and back, I was consulted next morning, and found the patient in the following condition : The teeth of the upper and lower jaw were firmly pressed together, so that I was only able with an effort to get the handle of a spoon between them. The expression of the face was grinning, as in risus sardonicus, through distortion of the muscles of the angles of the mouth. Swallowing was very difficult ; speech stammering. From the mouth saliva constantly dribbled. The pupils of both eyes were very much contracted. At times there were very painful convulsions in all the muscles, so that the whole body was as rigid and immovable as a statue. During these convulsions breathing was very difficult and entirely superficial. About thirty such convulsions occurred in the first twenty-hour hours, though of different duration and intensity. After these symptoms, it could not be doubted that we had to deal with traumatic tetanus, which had been occasioned by cold. The patient, for the first fourteen days, was put upon fifteen grains of bromide of potash and fifteen grains of chloral alternately every hour, so that he took 180 grains a day of each. From the second day of the treatment the patient fell into a slumberous condition, while at the same time a diminution in the frequency and violence of the convulsions was noticeable. After fourteen days opisthotonos completely disappeared, and there only remained for some days longer a waxy rigidity of the chin muscles. From this time on the chloral was continued in same doses four weeks longer. On the forty-eighth day after being taken sick the patient was dismissed from treatment as completely cured, but for six months general weakness of the body and inability to work persisted.—*Medical and Surgical Reporter.*

**Hydroquinone.**—Dr. Sylvester and Dr. Piccini, his pupil, have published (*Il Morgagni Revue de Therapeutique*, July 15, 1887) some recent observations on hydroquinone. This chinoline derivative was brought forward three years ago as a substitute for quinine, and very sanguine expectations of its therapeutical utility were entertained. Its antipyretic power was not questioned, but it was soon perceived that certain unpleasant, even dangerous conditions, were induced by it. Profound depression, severe rigors, profuse sweats, so often occurred when its antipyretic powers were utilized, that soon it ceased to be employed, and the safer antipyretics as antipyrin, acetanilide, salol, etc., substituted.

Professor Silvestrini and his pupil have, however, arrived at different conclusions from those heretofore held, and assert that it has an immenee superiority over its cogeners, in its perfect innocuousness in the strongest doses. It is prompt in action, and the higher the febrile temperature, the more powerful as an antipyretic. In typhoid, acute rheumatism, and erysipelas, it acts in a highly efficient manner ; and besides abating the temperature of fever, it has the power to remove the attendant symptoms—the disturbances of pulse and of respiration, the elimination of urea, the blood pressure, etc.

Hydroquinone is not irritant, and any gastro-intestinal trouble present is not increased by it. Having the antiseptic and germicide powers belonging to the group, when introduced into the intestinal canal it arrests the process of fermentation by inhibiting the microbes necessary to the process.

The dose of hydroquinone ranges from five to thirty grains, the frequency of administration determining to some extent. It is freely soluble in water, and unirritating, and hence can be given hypodermically.

**Boracic Acid Packing in Leucorrhœa.**—Dr. N. F. Schwartz recommends in the *St. Louis Courier of Medicine* the employment of dry packing of the upper vagina with boracic acid. He describes his manner of applying the tampon and powder as follows: Having first irrigated the vagina with water at as high a temperature as can well be borne by the patient, a cylindrical speculum is introduced, and the vaginal walls very carefully dried—first, with a soft sponge, and then with absorbent cotton. This done, boracic acid is poured into the mouth of the speculum, and pushed up against the uterus and vault of the vagina with a clean cork caught in a uterine sponge-carrier, sufficient acid being used to surround and bury the intravaginal portion of the cervix, filling the upper part of the vagina. A tampon of absorbent cotton is then firmly pressed against the packing, and held *in situ* until the folds of the vaginal walls close over it as the speculum is withdrawn. This should be allowed to remain three or four days, or even longer. A second, or even a third repetition may be necessary; but in none of my cases, numbering nearly a score, have I found more than a second packing called for, and in many one sufficed; and in no instance has its use occasioned pain, not even inconvenience.—*Medical Record.*

**Salicylate of Ammonium as an Antipyretic.**—Dr. Sullivan, of Brooklyn, N. Y., writes the *Journal of the American Medical Association:* Ammonium salicylate is certainly a very effective antipyretic. It will not reduce temperature as rapidly as antipyrin or antifebrin, but the antipyretic effect is more lasting than that produced by either of these agents.

In certain diseases of septic origin it exerts a curative action by tending to retard, and possibly inhibit, the development of septic elements in the system.

I cannot agree with Dr. Barnett that it is stimulating. From my own experience I am inclined to believe that in large doses, or in moderate doses continued for a long time, it has a decidedly depressing effect upon the heart and respiration ; but this depression may be obviated by administering it in combination with the aromatic spirits of ammonia.

It has an irritating action upon the kidneys, and consequently should not be given in scarlet fever, or in any case in which these organs are not in a healthy condition.

It has been my custom to prescribe it in from gr. viij–x doses every two to four hours during the first day, then at longer intervals as the requirements of the case may indicate. In some cases, ʒss given in divided doses during twenty-four hours will produce decided ringing in the ears ; others will take ʒj in the same time with little disturbance. To children about three years of age I usually administer it in gr. iij doses every four hours.

**Treatment of Ruptured and Divided Tendons.**—Dr. C. H. Wilkin presents a table of thirty-two unpublished cases, of which twenty-eight were treated by suture, with good results in twenty-two and benefit in all. He considers the injury in two classes, simple and compound. While in the former almost all authorities seem to agree that rest and position are sufficient, yet it would seem that a better result and certainly closer apposition could be obtained by the use of the suture ; of the propriety of the suture in the compound variety, there can be no question. The plan of treatment recommended, is first thorough cleansing of the part and irrigation of the sheath of the tendon with a 1–1,000 bichloride solution—except when the knee joint is involved, when it should be 1–5 000—by means of a small English catheter with a syringe attached, cocaine anæsthetic, and the use of silk-worm gut suture, two sutures being necessary in the average tendon, one being carried transversely through the tendon and the other antero posteriorily.—*New York Medical Journal.*

**Treatment of Otorrhœa.**—H. Knapp, of New York, in acute cases of otorrhœa, uses boracic acid as an antiseptic and cleansing the ear with the syringe three times a day. The powder is then introduced by means of a spoon until the canal is loosely filled. If the powder becomes moist, the patient is directed to syringe the ear and renew the application. The majority of acute cases do not require any other treatment. In chronic cases, he removes any polypoid growths or any curious bone that may be present, and then uses alcohol in fifty or sixty per cent. strength, or absolute, with sulpho-carbonate of zinc, and changes this with nitrate of silver. He continues this treetment until the ear is dry and there is no discharge, and directs the patient to do nothing beyond using a light cotton plug to filter the air. No attention is given to the perforations except in case a perforation of moderate size has perfectly clear edges, and remains in the same condition for weeks or months. Here he pastes a small piece of sized paper over the perforation. In many cases the hearing is improved. and it seems to stimulate the healing of the pe.foration.—*Med. Times*

**A New Hypnotic.**—Mering announced before the Strasburg Meeting of Neurologists the discovery of a new hypnotic, viz., hydrate of amylen, which represents a tertiary amylic alcohol. The drug has a specific gravity of 0.8, is little soluble in water, but readily so in alcohol. Mering tested the hypnotic power of the new remedy in sixty different cases, giving it altogether 250 times in paralysis, mental affections, insomnia caused by nervous excitation, and some cases of infectious fevers. The dose of hydrate of amylen is from 45 to 75 grains. The sleep induced by it lasts six to eight hours. The drug has a more pleasant taste than paraldehyde, and produces no after-effects. A convenient form of its administration is the following mixture:

℞. Amylen hydrat.............................grs. lx,

Aq. dest.....................................f ℥ j,

Extr. glycyrrh...............................f ʒ j,

S.—Shake well before taking.

—*Munchener Med. Wochenschrift*, June 21, 1887.

**Instantaneous Cure of Rheumatical Lumbago.**—Professor Germain See relates a case of rheumatical lumbago treated by him at the Hotel Dieu, (Salle Augustin, bed 18) in which an instantaneous cure was made by instantaneous injection of 8 grains of antipyrin. The man had suffered constantly for four days before treatment and was not able to move from the bed. The lumbago disappeared with the very first injection and never returned, though the remedy was continued for a few days as a precaution against relapse. In other cases of articular rheumatism (of the the fingers, toes, etc.,) the remedy, while not instantaneous, was rapid and sure. [Some years ago a patient of mine, Maj. John T. Burns, ex comptroller of the State of Georgia, after suffering for three days with rheumatical lumbago, was instantly and permanently relieved by two hypodermic injections of bisulphate of quinine. each injection containing 8 grains of salt.—F. L. J., in *St. Louis Medical Journal.*]

**Bromide of Ethyl.**—The value of bromide of ethyl as an anæsthetic in minor surgical operations, has recently been the subject of an address by Dr. Pauschinger (*Munch. Med. Wochenschr*). The conclusions drawn by him from the results of several trials was that the compound presents important advantages:

1. Because its freedom from danger allows of its use in minor, though painful operations, in which the use of chloroform would not be justifiable.

2. Its easy toleration, which spares the patient the disagreeable phenomena attending the chloroform narcosis.

3. Its convenient management which makes it possible for a practical operator to use it in cases of need without an assistant. These advantages are combined with a rapid and effective anæsthetic action.—*The Pharmaceutical Joarnal and Transactions.*

**Antipyretics.**—Dr. William Pepper, of Philadelphia: I have used both antipyrine and antifebrin largely in a variety of cases, and have not seen any of the dangerous symptoms which have been mentioned by some writers In the sudden acute pneumonias of children, with a temperature of 106° or 107°, with initial convulsions, where the prompt and repeated use of cold baths has been without gratifying results, full doses of either of these antipyretics have produced a remakably successful effect. As to the ability of these agents to replace cold baths under all circumstances, the evidence is not adequate. In the sudden hyperpyrexia occurring in the course of rheumatism, I am not prepared to accept the view that these drugs are capable of replacing the cold bath.—*Polyclinic.*

**Eucalyptus in Whooping-Cough and Bronchitis.**—Witthauer (*Memorabilien*, September 3, 1887) strongly recommends the administration of eucalyptus for whooping-cough and bronchitis. In whooping-cough he gives from five to twenty drops of a mixture of equal parts of tincture of eucalyptus and glycerine, according to the age of the child, every three hours. He also administers inhalations as follows: A piece of linen is made into a bag, in which is placed a piece of cotton on which ten drops of oil of eucalyptus is put every morning. The bag is tied around the neck of a child so as to be under the shirt, and so the child is in an atmosphere of eucalyptus by day and by night.

In bronchitis, Witthauer gives to an adult from fifteen to twenty drops of the tincture of eucalyptus every three hours. Inhalations are given by placing ten drops of the oil in a cup of hot water, and causing the steam to be inhaled.—*Allg. Med. Central Zeitung.*

**Effect of Carbolic Acid on Temperature.**—Dr. H. A. Hare draws the following conclusions from a number of experiments:

1. Carbolic acid possesses considerable power in lowering normal bodily temperature.

2. It possesses more influence over pyretic temperature than does salicylic acid, generally preventing a rise or causing a fall of temperature, but sometimes failing to do so.

3. Carbolic acid probably decreases arterial pressure when lowering temperature.

4. That its mode of decreasing normal bodily temperature is as yet not fully undersrood, although it would seem probable that it acts on both heat functions.

5. When influencing bodily heat in fever it acts chiefly by decreasing production, although it affects both functions.—*Therapeutic Gazette*, August 15, 1887.

**Antipyrine Hypodermically as a Substitute for Morphine.**—In a communication to the Academie des Sciences M. Germain See insists upon the advantages of antipyrin employed s multaneously as an anodyne in the place of morphine. Seven and a half or eight grains of antipyrine, dissolved in as many minims of distilled water, is quite sufficient, says the author, to quiet pain from whatever cause it may arise. The injection causes some pain, but this rapidly vanishes. M. See has used the remedy with uniform success in neuralgia, acute inflammatory rheumatism, the fulgurating pains of ataxia, hepatic and nephritic colic, anginas of the chest and asthmatic oppressions. In short, there exists no form of pain relievable by morphine which cannot be subdued equally as well and rapidly by antipyrine.—E. L. J., in *St. Louis Medical Journal.*

**The Toxical Properties of Cocaine.**—M. Bignon states the following results of numerous experiments on dogs and men: 1, cocaine produces physiological effects only in doses of 6–10 grs. given in 1-grain doses hourly; 2, its principal action refers to renal secretion, which it diminishes, accelerating at the same time the elimination of oxydized substances; 3, in higher doses it produces anuria and uræmic symptoms; 4, the paralytic action on the kidneys mostly appears within two to three hours; it is followed by diuresis proportionate to the preceding anuria; 5, cocaine acts toxically in an indirect way, only, that is, in cases the accumulation of toxical substances contained in the urine, resulting from the anuria, reaches a dimension sufficient to produce uræmia; 6, although the uræmic symptoms promptly diminish with diuresis being effected, the stimulating effects have a longer duration—about twenty-four hours.—*Bull. Gen. de Ther.*

**We learn** from the *Australasian Journal of Pharmacy* that charcoal given in water is an antidote in strychnine poisoning. The writer says he has had many of his dogs poisoned, but he did not care how bad they were if they were only alive, as a dose of charcoal would enable them to walk in an hour.—*New York Medical Times.*

**A Solvent for Antifebrin.**—Dr. Proegler, of Fort Wayne, Ind., states that he has used the aromatic spirits of ammonia as a solvent for antifebrin, and has found it very good for that purpose. It was suggested to him by Mr. Louis Schmidt, a pharmacist of that city.—*Medical Review.*

**Antipyrine, Hypodermically, instead of Morphine.**—Prof. See advises hypodermic injections of antipyrine ($7\frac{1}{2}$ grains dissolved in water) in painful conditions, as a substitute for morphine. He says:

"The subcutaneous injection, which is made just as in the case of a morphine injection, produces, after a painful sensation of tension, which lasts a few moments, a considerable remission of pain, whatever may be its cause. On comparing the effects with th se of morphine, it is easy to note that antipyrine, given in hypodermic injections, does not present any of the disadvantages almost constantly produced with the former, such as vertigo or vomiting, neither does it throw the patient into a state of somnolency, nor produce that artificial excitation which eventually leads to morphinomania. Lastly, and this is most important, it adds to its sedative action a curative effect which is not possessed in any case by morphine."

He mentions its use particularly in acute rheumatism, neuralgia, hepatic and renal colic, angina pectoris, etc.—*The Medical World.*

**Ergot Again.**—Dr. Star (*Medical World*) says of ergot: During twenty-eight years I have had recourse to its contractile power on the uterus. Do not consider that it can originate labor when given in proper doses. Rarely use it singly. In combination with fl. ext. of gelsem., I find it gives great steadiness to the uterine contractions.

℞. Fl. ext. ergot.............................gtts. xxxv,
fl. ext. gel..................................gtts. x.

The ergot may be repeated oftener than the gel. This frees the system from irregular and neuralgic pains concentrating in the uterus.

When the head of the child is large, and the lapping of the cranial bones do not readily take place, I give from 1 to 2 grains of opium, and it really seems to aid the laboring lady, so that I generally meet the approving smile of the newly made mother for the happy results of my therapeutics. I never give, during a labor, more than three combined doses of ergot and gelsem., nor more than two anodyne relaxant doses.

Ergot and gelsem., with opium, before delivery, are mv best medicines to prevent hemorrhage and shock. Would fear the use of chloral hydrate, lest unexhausted chloral in the blood, after labor was completed, should over-act on the brain, producing weakness of the heart's action.

**Fowler's Solution for Nervous Condition of Menopause.**—Dr. White says: In October number of *The World*, Dr. W. R. Godfry wishes to know if there is such a thing known as a remedy for "hot flashes that are so troublesome to ladies during change of life." I would say that there is no specific for this unpleasant difficulty, at least I know of none. But having seen Fowler's Solution from 3 to 5-drop doses three or four times a day, recommended in one of my medical journals a few years since, for the nervous symptoms accompanying the period of the menopause, I can truly bear testimony to its good effects in several cases for which I have prescribed it, and would say to Dr. Godfry that I think it a remedy well worthy of trial in those cases.—*Medical World.*

**Ergot in Ophthalmic Practice.**—Thinking my experience with ergot in eye diseases might be of interest to readers of the *Journal.* I will give in brief my use of the drug. In catarrhal ophthalmia (muco-purulent conjunctivitis), I use:—

℞. Acid boric.................................gr. ij,

Lloyd's ergot...... ..........................ʒ ss to ℥ j,

Aqua dest., q s.................................℥ iv.

M. Ft. Col. Sig.—Two drops in the eye four or five times a day.

Relief from the smarting pain is experienced almost immediately, and the inflammatory action cut short.

In granular conjunctivitis especially do we find the above prescription valuable, except in cases of long standing, when I first use either a 4-per cent. solution of nitrate of silver, or squeeze the lids in order to evacuate the glands, after which the ergot and boric acid are used.

Pinguecula is another morbid condition which may be benefited by the use of ergot, and I have used it successfully in a number of cases where the parties objected to the simple operative measures required for the removal of this nuisance.

In ophthalmia neonaotrum the ergot has, in my hands, given negative results until after the purulent character of the discharge had disappeared, then the effects were pleasant, and served to hasten recovery.

Some may ask whether the boric acid is not the active agent. I say no, at least so far as my experience goes, as I have used both the acid and ergot separately on the same and different individuals, with the uniform result of most rapid improvement when the ergot was employed. However, the action of the two agents combined is more rapid than that of either one singly.

The good results from the use of ergot in eye troubles led me to try its virtues in that troublesome aural affection, chronic suppuration of the middle ear. Having several cases on hand that would not yield to any treatment known in aural therapeutics, and becoming disgusted, as well as the patient, as a last resort I commenced using ergot in the following form:

℞. Fluid extract ergot. ...........................fʒ ss,

Acid boric...................................ʒ j.

In this case the ergot preparation *must* be free from glycerine, otherwise a dry powder will not result. After the ear is thoroughly cleansed and dried, pack the powder in the canal quite firmly, and allow it to remain about three days, if the discharge does not interfere, then the process is to be repeated. In four cases which had resisted all other forms of medication, favorable progress is now being made. In this class of cases, only repeated experiments will establish the value of ergot as a therapeutic agent.

In both ophthalmic and aural diseases, it is of course supposed that proper constitutional treatment is followed, for reliance on local measures alone will usually prove very unsatisfactory.

In granular and catarrhal conjunctivitis, I have used the ergot and boric acid on over two hundred cases, with such uniformly good results that I do not think of employing any other local remedial agents for those diseases.—*Dr. Foltz, Cincinnati Eclectic Medical Journal.*

**Typhoid Fever.**—Professor Waugh (*in College and Clinical Record*) thus summarises the treatment of typhoid fever: "I have no faith whatever in specifics, but set a high value upon the observance of strict hygiene, insuring absolute mental and physical quiet to the patient, keeping the room well ventilated and comfortable, prescribing a suitable diet, treating the symptoms as they arise, and possibly administering the benzoate of ammonium throughout the disease, in the dose of 20 grains every four hours.

**How to Preserve the Dead Body.**—Dr. H. Speier, of Duluth, Minn., writes as follows to the *Pharmaceutical Record:* "I will briefly give the method for preserving bodies invented a few years ago by Wickersheim, of the Anatomical Museum of Berlin. The process is simple and cheap, and the inventor claims that by it the color, form and flexibility of dead animal bodies and all their tissues are preserved. He says that after several years sections for scientific or legal purposes can be made, decomposition and even foul odor being entirely absent. The preserving fluid is made as follows: Take of potash (caustic potassa), 60 parts; arsenious acid, 10 parts; dissolve by heat in 500 parts of water, then add enough water to make 3,000 parts, in which dissolve alum 100 parts; salt, 25 parts; saltpetre, 12 parts. After cooling, filter the solution. To 10 litres of glycerine and 1 litre of methyl-alcohol. Inject the body with the preserving fluid, then immerse it in the same for a few days. Rub and dry the body, and envelop it in a sheet soaked with the same fluid, and place in an air-tight receptacle."—*College and Clinical Record.*

**Cocaine Internally.**—Dr. L. Frey, of Bekes, having as a patient a young woman who had mitral insufficiency and hypertrophy of the heart associated with hyperæsthesia of different parts of the body, which caused extreme irritability of the stomach and constant vomiting, so that for some days she had scarcely twenty minutes' intermission, tried digitalis, opiates, ice, cold applications, etc., but without any effect. He then determined to try cocaine internally. He gave three-quarters of a grain dissolved in water, which was followed by a cessation of the attacks of vomiting for two hours; another dose gives the patient six hours' rest, after which another violent attack came on. The third dose stopped the vomiting altogether, after which all the other symptoms from which the patient suffered, rapidly improved.—*The Lancet, August 6, 1887.*

**Castration of Criminals** —The following is the recommendation of an enthusiastic sociologist who proposes castration as a means of limiting crime. The good effect of this kind of punishment upon the criminal class would be fourfold.

1. No offspring with an inherited tendency to commit crime.
2. An added terror to the punishment inflicted for breaking the laws.
3. A gradual improvement in time of the morals of the public at large.
4. An improvement in the disposition of the person operated upon.—*Medical News.*

**New Remedies for the Morphine** Habit.—Dr. Oscar Jennings (*The Lancet*) announces the discovery of certain drugs, which, he says, will enable a person who is desirous of leaving off the morphine habit to carry his intentions to a successful issue. He describes the habit as possessing an organic distress, and cerebral craving, the first of which is relieved by *sparteine*, and the second by *glonoin*, from which drugs the patient may get relief from his craving, until the system has accommodated itself to the absence of morphine.—*American Medical Journal.*

**Succedaneum of Morphia.**—In a highly important memoir, presented to the "Academi des Sciences" in its meeting of July 11, 1887, Dr Germain See points out the efficiency of antipyrine against most of the affections which at the present time lead to the so disastrous use of morphia. The renowned physician shows in the new substance, a means of preserving from chronic poisoning, the numerous patients who seek for an alleviation of the sufferings in morphia.—*La Nature.*

**Seven Consecutive Cases of Charbon Treated Successfully by Excision** (Grey's Hospital. Mr. Bryant).—The cases in detail presented nothing of especial interest. All were well marked cases of charbon. The method of operating was to carry the excision into healthy tissue entirely beyond the diseased structures, and to apply to the surfaces either pure carbolic acid or the actual cautery. Dry dressings were used. All the cases recovered.—*Medical Times.*

**Eucalyptus Honey.—A** small black bee makes its home in the eucalyptus tree and produces a large amount of honey, which has a strong flavor of the eucalyptus. When mixed with water or milk it forms a pleasant drink and diffuses a feeling of warmth through the body, the voice becomes clearer and the lungs seem stronger. It has been found a very pleasant and effectual remedy in bronchitis and laryngeal affections.—*Medical Times.*

**Quinine.**—In giving quinine, it is well to combine with hydrobromic acid; it renders the disagreeable cerebral effects much less, does not interfere with its action, renders it more soluble, and adds to its efficacy.—*College and Clinical Record.*

